# Urban *versus* rural lifestyle in adolescents: associations between environment, physical activity levels and sedentary behavior

**DOI:** 10.1590/S1679-45082016AO3788

**Published:** 2016

**Authors:** Manuela Ferreira Regis, Luciano Machado Ferreira Tenório de Oliveira, Ana Raquel Mendes dos Santos, Ameliane da Conceição Reubens Leonidio, Paula Rejane Beserra Diniz, Clara Maria Silvestre Monteiro de Freitas

**Affiliations:** 1 Universidade de Pernambuco, Recife, PE, Brazil.; 2 Centro Universitário Asces-Unita, Caruaru, PE, Brazil.; 3 Faculdade Boa Viagem, Recife, PE, Brazil.; 4 Universidade Federal de Pernambuco, Recife, PE, Brazil.

**Keywords:** Motor activity, Adolescent behavior, Sedentary lifestyle, Urban population, Rural population

## Abstract

**Objective:**

To analyze the levels of physical activity and sedentary behavior in adolescents living in urban and rural areas.

**Methods:**

An epidemiological, cross-section study with quantitative design, carried out at the regional level. The sample comprised 6,234 students aged 14 to 19 years, selected using random cluster sampling. The χ^2^ test and binary logistic regression were used in the analysis.

**Results:**

A total of 74.5% of adolescents lived in urban areas. After adjustment, rural residents spent less time watching television (odds ratio – OR: 0.45; 95% confidence interval – 95%CI: 0.39-0.52), using a computer and/or playing video games (OR: 0.30; 95%CI: 0.22-0.42), or sitting down (OR: 0.66; 95%CI: 0.54-0.80); chose passive leisure less often (OR: 0.83; 95%IC: 0.72-0.95) and were less likely to be classified as insufficiently active (OR: 0.88; 95%IC: 0.78-0.99) when compared to urban residents, regardless of sex or age. The fact that adolescents living in rural areas who did not work were more likely to be classified as insufficiently active (OR: 2.59; 95%CI: 2.07-3.24) emphasized the significant role of occupation in physical activity levels in this group.

**Conclusion:**

Adolescents living in rural areas were less exposed to the sedentary behaviors, chose more active leisure, and had higher levels of physical activity. Place of residence and occupation may play a major role in youth lifestyle.

## RESUMO

**Objetivo:**

Analisar os níveis de atividade física e o comportamento sedentário em adolescentes das áreas urbanas e rurais.

**Métodos:**

Estudo epidemiológico, transversal, com abordagem quantitativa e abrangência estadual, cuja amostra foi constituída por 6.234 estudantes (14 a 19 anos), selecionados por meio de uma estratégia de amostragem aleatória de conglomerados. As análises foram realizadas por meio do teste χ^2^ e da regressão logística binária.

**Resultados:**

Na amostra, 74,5% dos adolescentes eram residentes em área urbana. Após o ajuste, constatou-se que os adolescentes oriundos da área rural usavam menos televisão (*odds ratio* – OR: 0,45; intervalo de confiança de 95% – IC95%: 0,39-0,52), computador e/ou videogame (OR: 0,30; IC95%: 0,22-0,42), passavam menos tempo sentados (OR: 0,66; IC95%: 0,54-0,80), optaram menos pelo lazer passivo (OR: 0,83; IC95%: 0,72-0,95) e tinham menos chances de serem classificados como insuficientes ativos (OR: 0,88; IC95%: 0,78-0,99), quando comparados àqueles que residiam na área urbana, independentemente do sexo e da idade. Os adolescentes da área rural que não trabalhavam apresentaram mais chances de serem classificados como insuficientemente ativos (OR: 2,59; IC95%: 2,07-3,24), mostrando que a ocupação tinha um papel importante no nível de atividade física deste grupo.

**Conclusão:**

Os adolescentes residentes na área rural estiveram menos expostos aos comportamentos sedentários, optaram mais por um lazer ativo e apresentaram um melhor nível de atividade física, podendo a zona de domicílio e a ocupação influenciar no estilo de vida deles.

## INTRODUCTION

Adolescence is characterized by biological, physical, psychological and social changes, with potential direct impacts on daily activities.^(1)^ The number of youth who do not comply with the World Health Organization recommendations on daily physical activity is on the rise.^(2-4)^ Several factors may account for this scenario, such as time spent on electronic devices,^(5)^ passive travel to school,^(6)^ lack of Physical Education in schools,^(2)^ limited access to settings providing opportunities for physical activity,^(2)^ lack of maternal physical activity^(7)^ and low schooling and income levels.^(8)^ These are alarming facts, which may be related to developing chronic degenerative disease and mortality risks.^(9,10)^

Studies with different designs suggest that both the level of physical activity and fitness of youth and adults are related to, or may be influenced by, the environmental context in which they live.^(11)^ Given the environment is a determining factor of lifestyle, people living few kilometers apart, in the same geographical area, may have different lifestyles when it comes to physical activity, particularly when rural and urban areas are compared.^(12)^ Greater availability of equipment and public leisure spaces in urban areas, such as squares, courts, pedestrian boulevards and bike paths, may be associated with high levels of physical activity.^(5,13)^

Urban and rural areas may be associated with two different lifestyles^(14)^ and environment characteristics may contribute to lower levels of physical activity and fitness in adolescents.^(4,8)^ However, studies addressing physical activity levels and sedentary behavior in adolescents living in rural areas are scarce, and few studies control for these variables in the analysis. Such data may be used to plan interventions aimed to promote healthier habits among adolescents, in an effort to reduce health problems in adulthood, given the associations between diseases and risk behaviors at a younger age.^(4,15)^

## OBJECTIVE

To analyze the levels of physical activity and sedentary behavior in adolescents living in urban and rural areas, in the light of socioenvironmental characteristics.

## METHODS

This is a descriptive study with quantitative design involving cross-sectional school-based epidemiological surveys at the municipal level. The study sample comprised male and female students aged 14 to 19 years enrolled in public high schools, in the State of Pernambuco, Brazil. Improperly filled out questionnaires, students who were absent at the time of data collection or those who refused to participate were excluded. The survey was entitled “Physical activity practice and health risk behaviors in high school students in the State of Pernambuco: a temporal trend study”.

Two-stage cluster sampling was used in this study. In the first sampling stage, schools were randomly selected according to size and geographical location to serve as sampling units. Groups of students were then drawn according to school hours and grades in the selected schools.

A pilot study was conducted to test the applicability of the instrument prior to data collection. Data were collected at a randomly selected reference public school in the city of Recife (PE), from a sample comprising 86 adolescents.

Reproducibility indicators had moderate to high intraclass correlation coefficients for variables employed in this study. Agreement coefficients (kappa index) were as follows: 0.78 for watching television; 0.62 for playing videos games and/or working on a computer; 0.44 for time spent sitting down (screen time excluded); 0.67 for favorite leisure activity; 0.59 for level of physical activity and 1.00 for place of residence.

Data were collected during the first (May and June) and second (August through November) terms of 2011. Adolescent classification as urban or rural residents was based on self-reported place of residence in a previously tested, self-administered translated version of the Global School-Based Student Health Survey (GSHS), proposed by the World Health Organization. The following domains were used: “personal information”, “physical activities” and “behavior at home and in school”.

The variables associated with sedentary behaviors were “time spent watching television”, determined by calculation of the weighted mean of the answers given to the following questions: “On school days (Monday through Friday), how many hours do you spend watching television per day?”, and “On weekends (Saturday and Sunday), how many hours do you spend watching television per day?” (question 1x5 + question 2x2)/7.

The variable “time spent on a computer and/or playing video games” was determined by the weighted mean of the answers given to the following questions: “On school days (Monday through Friday), how many hours do you spend on a computer and/or playing video games per day?”, and “On weekends (Saturday and Sunday), how many hours do you spend on a computer and/or playing video games per day?”.

The variable “time spent sitting down (screen time excluded)” was investigated by calculating the weighted mean of the answers given to the following questions: “On school days (Monday through Friday), how many hours do you spend sitting down chatting with friends, playing cards or dominoes, talking on the telephone, commuting as driver or passenger, reading or studying (time spent watching television or using a computer excluded)?”, and “ On weekends (Saturday and Sunday), how many hours do you spend sitting down chatting with friends, playing dominoes or cards, talking on the telephone, commuting as driver or passenger, reading or studying (time spent watching television or using a computer excluded)?”. Sedentary behaviors were classified as less or more than 4 hours of exposure.

As regards the variable “level of physical activity”, two GSHS questions were considered: “During the last 7 days, how many days were you physically active for a total of at least 60 minutes per day?” and “In a typical or usual week, how many days are you physically active for a total of at least 60 minutes per day?”. Physical activity level assessment (questions 1 and 2) was based on the formula suggested by Prochaska et al.^(16)^: (question 1+ question 2)/2. Adolescents achieving values lower than 5 days were considered insufficiently active (*i.e.*, non-compliant with physical activity recommendations). The favorite leisure activity was divided as active (sports, physical exercise, swimming or bicycling) or passive leisure (playing dominoes or cards, watching television, playing video games, using computers or chatting with friends) as performed in previous study.^(6)^

Data were tabulated using EpiData (version 3.1) and electronically controlled data entry. Double data entry verification was used to ensure data entry consistency. Typing errors were tracked using the VALIDADE tool and corrected. Data analysis was performed using software (Statistical Package for the Social Sciences, SPSS; version 10.0 for Windows).

Inferential and descriptive statistic procedures were used. Absolute and relative frequency distributions were constructed in descriptive analysis. The Pearson’s χ^2^ test was used in inferential analysis to investigate isolated associations between level of physical activity and place of residence (urban or rural area), and to investigate variables included in the model, explore potential confounding factors and check the need for statistical adjustment in the analyses. Multivariate analysis was based on binary logistic regression; odds ratio (OR) estimates and 95% confidence intervals (95%CI) were used to express the degree of association between dependent (level of physical activity, sedentary behavior and favorite leisure activity) and independent variables (place of residence), with adjustment for potential confounding factors (sex, age, occupation and maternal schooling). Interactions between level of physical activity, place of residence and gender were tested following the determination of variables predicted in the final model.

Confounding variables were introduced simultaneously (Enter method) and only those with significance levels below 0.20 (p<0.20) retained. Only significant variables were included in the final regression model. Results were presented as crude and adjusted OR values, and 95%CI; results were considered significant when p<0.05.

This study was approved by the Ethics Committee for Research Involving Humans, of the *Universidade de Pernambuco*, protocol number 159/10, CAAE: 0158.0.097.000-10. An Informed Consent Form was signed by parents of minors and by students aged over 18 years.

## RESULTS

**Figure 1:**
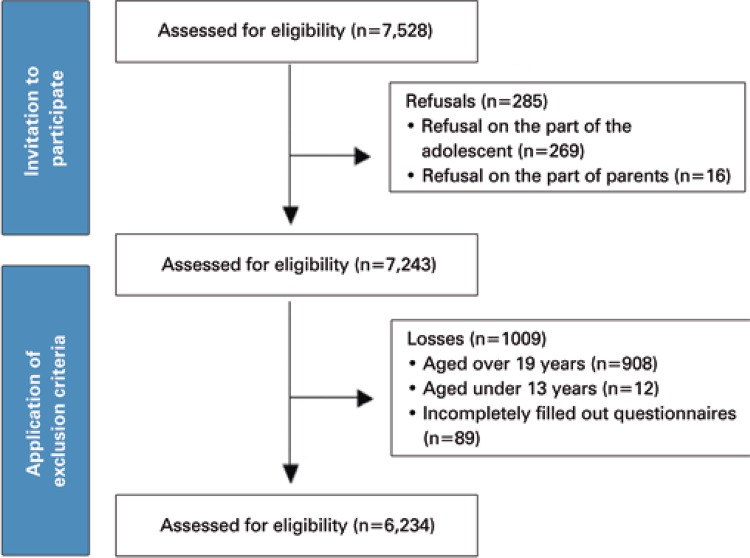
Flowchart representing the inclusion of students of the State of Pernambuco in the study

Eighty-five schools in 48 cities within the State of Pernambuco were visited. The data collection flowchart is presented in figure 1; the final sample comprised 6,234 adolescents aged 14-19 years, of which 59.7% were girls. Overall, 53.4% adolescents were aged between 16 and 17 years, and 74.5% lived in urban areas. Of those living in rural areas, 28.3% worked (Table 1).

**Table 1:** Characteristics of adolescents living in urban and rural areas

Variables	Urban (n=4,646)	Rural (n=1,588)	Total (n=6,234)	p value
	n (%)	n (%)	n (%)	
Sex				
Male	1,878 (40.4)	640 (40.3)	2,524 (40.3)	0.938
Female	2,766 (59.6)	947 (59.7)	3,737 (59.7)	
Age, years				
14-15	983 (21.2)	357 (22.5)	1,350 (21.6)	0.099
16-17	2,554 (55.0)	779 (49.1)	3,345 (53.4)	
18-19	1,109 (23.8)	452 (28.4)	1,569 (25.0)	
Occupation				
Works	938 (20.2)	448 (28.3)	1,390 (22.3)	<0.001
Does not work	3,697 (79.8)	1,135 (71.7)	4,857 (77.7)	
Maternal schooling, years of education				
>8	1,669 (41.2)	228 (17.2)	1,903 (35.3)	<0.001
≤8	2,378 (58.8)	1,100 (82.8)	3,491 (64.7)	

Students living in rural areas had healthier habits as compared to those living in urban areas, as shown by preference for active leisure activities (43.2% *versus* 39.5%), shorter sitting down time (90.1% *versus* 83.7%), less computer and/or video game exposure (97.1% *versus* 88.1%), less television exposure (88.7% *versus* 86.0%) and higher levels of physical activity (37.3% *versus* 34.5%) (Table 2).

**Table 2:** Level of physical activity and sedentary behaviors among adolescents living in urban and rural areas

Variables	Urban (n=4,646)	Rural (n=1,588)	Total (n=6,234)	p value
	n (%)	n (%)	n (%)	
Level of physical activity				
Active	1,596 (34.5)	591 (37.3)	2,192 (35.1)	0.041
Insufficiently active	3,031 (65.5)	992 (62.7)	4,047 (64.9)	
Time spent watching television, hours				
<4	3,988 (86.0)	1,404 (88.7)	5,420 (86.5)	0.006
≥4	647 (14.0)	178 (11.3)	826 (13.2)	
Time spent using a computer and/or playing video games, hours				
<4	4,081 (88.1)	1,537 (97.1)	5,647 (90.5)	<0.001
≥4	549 (11.9)	46 (2.9)	596 (9.5)	
Time spent sitting down (screen time excluded), hours				
<4	3,848 (83.7)	1,412 (90.1)	5,286 (85.4)	<0.001
≥4	749 (16.3)	155 (9.9)	907 (14.6)	
Favorite leisure activity				
Active leisure^*^	1,621 (39.5)	611 (43.2)	2,241 (40.4)	0.015
Passive leisure^†^	2,483 (60.5)	804 (56.8)	3,305 (59.6)	

* Active leisure: sports, physical exercise, swimming or bicycling; ^†^ Passive leisure: playing dominoes or cards, watching television, playing video games, using a computer or chatting with friends.

One model was constructed for each behavior. The variables were adjusted for sex, age, occupation and maternal schooling; only variables with p<0.20 were retained in the model. Adjustments revealed that adolescents living in rural areas had lower risks of exposure to computer and/or video games (OR=0.30; 95%CI: 0.22-0.42), television (OR=0.45; 95%CI: 0.39-0.52) and sitting down time excluding screen time (OR=0.66; 95%CI: 0.54-0.80).

As regards physical activity, adolescents living in rural areas were less likely of falling within the insufficiently active category, regardless of sex (OR=0.88; 95%CI: 0.78-0.99). However, this association became non-significant following adjustment for occupation (OR=0.94; 95%CI: 0.83-1.06) (Table 3). The investigation of associations between occupation and level of physical activity was limited to adolescents living in rural areas; the fact that the likelihood of being categorized as insufficiently active was higher among those who did not work (OR=2.59; 95%CI: 2.07-3.24) emphasizes the significant role of work in relation to physical activity among adolescents living in rural areas. No interactions were found between level of physical activity, place of residence and sex (0.21); therefore, sex stratification was not used in the analysis.

**Table 3:** Relationship between place of residence (urban or rural area), level of physical activity and sedentary behaviors

Variables	Adolescents living in rural areas					

	*Odds ratio*	95%CI	p value	*Odds ratio*	95%CI	p value
	(crude)			(adjusted)		
Level of physical activity^*^						
Active	1			1		
Insufficiently active	0.88	0.78-0.99	0.041	0.94	0.83-1.06	0.29
Time spent using watching television, hours^†^						
<4	1			1		
≥4	0.78	0.65-0.93	0.006	0.45	0.39--0.52	<0.001
Time spent using a computer and/or playing video games, hours^‡^						
<4	1			1		
≥4	0.22	0.16-0.30	<0.001	0.30	0.22-0.42	<0.001
Time spent sitting down (screen time excluded), hours^‡^						
<4	1			1		
≥4	0.56	0.47-0.68	<0.001	0.66	0.54-0.80	<0.001
Favorite leisure activity^§^						
Active leisure^¶^	1			1		0.008
Passive leisure^||^	0.86	0.76-0.97	0.015	0.83	0.72-0.95	

* Adjusted for sex and occupation; ^†^ adjusted for age and occupation; ^‡^ adjusted for sex, age, maternal schooling and occupation; ^§^ adjusted for sex and occupation; ^¶^ active leisure: sports, physical exercise, swimming or bicycling; ^||^ passive leisure: playing dominoes or cards, watching television, playing video games, using a computer or chatting with friends.

## DISCUSSION

Adolescents living in rural areas had higher levels of physical activity, showed less preference for passive leisure and were less exposed to sedentary behaviors compared to adolescents living in urban areas.

This more active lifestyle may be associated with participation in the labor market, consisting mainly of physical labor in subsistence agriculture^(17,18)^ and common household activities performed by women living in rural areas.^(19)^ This finding differs from other studies^(5,20)^ that reported higher likelihood of being classified as insufficiently active among adolescents living in rural areas, due to greater availability of public leisure spaces (squares, sport centers and public courts) in urban compared to rural areas. Hence, inclusion of adolescents living in rural areas evaluated in this study in the labor market was thought to be an important factor behind the higher levels of physical activity documented in this group.

Leisure-related physical activity is thought to be one of the most important dimensions of physical activity,^(17)^ given 55% to 65% of moderate to vigorous activities undertaken by children and adolescents fall within this category.^(9)^ This study revealed that youth living in rural areas tended to have lower preference for leisure activities involving lower energy expenditure, such as playing dominoes or cards, watching television, playing video games, using a computer or chatting with friends. Similarly, recent studies have documented high prevalence of such sedentary behaviors and low levels of physical activity among youth living in urban areas,^(21,22)^ which may reflect ease of access to technological devices, particularly computers.^(23)^ The fact that most youth who spend excessive amounts of time on a computer do not comply with recommended physical activity levels has also been reported,^(24)^ and suggests the replacement of physical activity for sedentary behaviors.^(17)^

Individual (motivation, self-efficacy, motor skills and other health-related behaviors) and environmental (access to labor or leisure spaces, costs, sociocultural support and limited time availability) characteristics may impact on physical activity levels and acquisition of sedentary behaviors.^(25)^ Time constraints are thought to be a major reason underpinning insufficient physical activity levels and/or sedentary behaviors. Still, this factor was not significant in this study, given youth living in rural areas were more physically active, even though they entered the labor market sooner. Physical Education lessons at school may also influence physical activity levels,^(4,26)^ as may parental behavior,^(27)^ bearing in mind that the family is the first learning environment of children and adolescents.

As regards parental influences, maternal schooling may play a role in youth lifestyle. Studies suggest maternal schooling (low to intermediate) is significantly associated with exposure to insufficient levels of physical activity and acquisition of sedentary behaviors.^(28,29)^ However, this study revealed that maternal schooling was lower among adolescents living in rural compared to those living in urban areas, even though the former were more phsically active and less exposed to sedentary behaviors. This may reflect the relation between low parental schooling and lower socioeconomic status^(30)^ and the resulting limited access to electronic devices (computer, video games and television), which would decrease the likelihood of adherence to sedentary behaviors among adolescents living in rural areas, and encourage higher levels of physical activity, in the form of greater use of active transportation means in daily commuting, as well as more participation in household acitvities, with expressive contributions to physical activity levels in this group.

The definition of urban and rural areas goes beyond simple spatial boundaries to include different social, economic and cultural organization forms, leading to two distinct lifestyles.^(14)^ In this context, some urban environment factors, such as lack of security, high population density and excessive involvement in intellectual activities may contribute to reduced level of physical activities and greater exposure to sedentary behaviors, resulting in less fitness among adolescents.^(6,9)^ Hence, place of residence may also be a significant factor in obesity development, a major global health issue directly associated with physical inactivity.^(12)^

Some limitations in this study must be highlighted. Cross-sectional design and the correlative nature of data prevent the determination of a causal relation between adolescents’ place of residence and habits. Furthermore, this school-based study did not include students enrolled in private schools, which precludes generalization of results to all adolescents within the State of Pernambuco. Self-reported physical activity represents yet another limitation; also, data on individual and environmental factors with directly or indirectly impacts on physical activity levels, such as social support, open spaces and time availability, were not collected.

Among the strengths of this study, the representative nature of the sample and the use of established sampling procedures to ensure the inclusion of students enrolled in different shifts and living and urban and rural areas, stand out.

Bearing in mind the importance of a healthy lifestyle in the quest for better quality of life and reduced incidence of diseases associated with low levels of physical activity,^(19)^ population-based studies are warranted to determine the prevalence of sedentarism and low levels of physical activity in urban and rural populations. Such studies could provide major contributions to proper planning and implementation of health promotion programs, encouraging regular physical activity practice, and fighting against sedentary behavior. They could also play a role in the development of public policies aligned with social, environmental and cultural aspects of different populations.

## CONCLUSION

The different characteristics of urban and rural environments were related to respective residents’ lifestyles. Adolescents living in rural areas were less exposed to sedentary behaviors, chose more active types of leisure and had higher levels of physical activity.
